# Sustaining substance use recovery housing for people taking medications for opioid use disorder: diverse funding, strategic partnerships, and charging rent

**DOI:** 10.3389/fpubh.2025.1528971

**Published:** 2025-04-30

**Authors:** I. Niles Zoschke, Kathryn R. Gallardo, Danielle Gillespie, Hannah L. N. Stewart, Serena A. Rodriguez, Sheryl A. McCurdy, J. Michael Wilkerson

**Affiliations:** ^1^Community Health Sciences Division, Alcohol Research Group, University of California at Berkeley, Berkeley, CA, United States; ^2^UTHealth Houston School of Public Health, Houston, TX, United States; ^3^UTHealth Houston School of Public Health in Dallas, Dallas, TX, United States

**Keywords:** MOUD, dissemination and implementation science, sustainability, treatment, maintenance, substitution, sober living homes, recovery support services

## Abstract

**Introduction:**

Opioid use disorder is a U.S. epidemic, and recovery housing plays a critical role by providing stable, supportive environments that promote long-term recovery. However, little is known about the sustainability of recovery homes, particularly those serving people taking medications for opioid use disorder.

**Methods:**

We applied thematic analysis to interviews with 29 staff and operators from 10 recovery homes serving people taking medications for opioid use disorder across five Texas cities.

**Results:**

Operators relied on diverse funding sources, leveraged strategic partnerships and professional certifications, and planned to charge rent when public funding ended. Staff and operators balanced financial sustainability with maintaining quality services.

**Discussion:**

Findings offer guidance for sustaining recovery homes that support medications for opioid use disorder. Recovery home operators can leverage professional networks, strengthen partnerships, rely on diverse funding sources, and reduce operational costs to sustain services. Policymakers can further support sustainability by establishing long-term funding mechanisms and reducing financial barriers to professional certification, ultimately improving service quality and access.

## 1 Introduction

Opioid use disorder (OUD) is a national epidemic, affecting more than seven million people in the United States (1). In 2023 alone, opioid overdoses were involved in an estimated 81,083 deaths (2). A range of recovery pathways have emerged to curb the OUD epidemic, including the use of medications for opioid use disorder (MOUD). These medications are effective in reducing overdose risk and preventing opioid-related injuries (3). However, despite their effectiveness, MOUD are not reaching many people who could benefit from them (4).

Recovery homes have also emerged as a major approach to supporting individuals with OUD and as a major asset for increasing MOUD access and receipt (5). Recovery homes provide safe, supportive, group-living arrangements for people in recovery (6). The National Alliance for Recovery Residences (NARR) is a nonprofit organization that establishes standards, promotes best practices, and supports the development of quality recovery housing across the United States. NARR established four classifications for recovery homes: Level I: democratically run, peer-led homes; Level II: appointed leaders or managers with set house rules; Level III: homes with trained and supervised staff, peer recovery support services and life skills development classes; and Level IV: homes that provide the services of a Level III home as well as treatment services (7). Unfortunately, recovery homes have not always welcomed people taking MOUD, and many still harbor stigma towards MOUD use, as more traditional approaches to recovery view it as inauthentic recovery (5, 8, 9). To combat stigma, the federal government and advocacy agencies have taken action to ensure recovery residences accept people taking MOUD. For example, the U.S. Department of Justice has deemed that refusing services to people on the basis that they take MOUD is against the law (10). Moreover, the National Alliance for Recovery Residences (NARR) has issued guidance to recovery home stakeholders for effectively supporting residents taking MOUD as part of their recovery, including proper screening practices that treat these residents fairly (11). The Substance Abuse and Mental Health Services Administration (SAMHSA) also recommends that recovery home operators should not prevent residents from taking MOUD as prescribed or bar prospective residents who take MOUD from moving in (12).

While little has been published on the effectiveness of recovery homes, and less so on the effectiveness of recovery homes for people taking MOUD, recovery homes do offer promise in the OUD continuum of care (6). For example, researchers have shown that people living in a recovery home for more than 6 months had lower rates of substance use at 24 months follow-up relative to referral to outpatient treatment, mutual aid groups, and other recovery resources (13). Other researchers have also confirmed that drug and alcohol use decline among people living in recovery homes at 6-month, 12-month, and 18-month follow up (14). Recovery homes also correlated with increased rates of employment for residents with extended stays (15) and pose a major alternative to incarcerating people found guilty of a drug use related offense (16).

Because recovery homes play a crucial role in supporting long-term recovery, their sustainability must be researched and understood. Sustainability, in this context, refers to maintaining the critical elements of a public health initiative or program over time to achieve ideal outcomes (17). While many public health initiatives and research studies focus on the implementation of services, less attention is paid to the sustainability of programs once funding diminishes (18). Proctor et al. (19) argue that despite major advancements in developing and implementing evidence-based, public health interventions, sustaining interventions is among the most critical concerns in modern research. Because few studies have researched the sustainability of recovery homes for people taking MOUD as part of their recovery pathway, greater emphasis on this topic is needed.

Recovery home sustainability and equitability faces significant challenges due to a lack of standardization, inconsistent funding, and gaps in research. One of the primary obstacles to sustainability is the absence of standardized services and limited financial support across the recovery home industry. Recovery residences are not federally regulated, leading to significant variability in operations (20). Many homes are privately owned and function independently of formal treatment facilities (21). Consequently, funding opportunities and approaches differ widely (22). Most residents must pay for services out of pocket due to the fact that third-party payer systems are uncommon (6), making these homes particularly vulnerable to economic shifts, such as those caused by the COVID-19 pandemic (23). Economic factors, including residents’ employment status and ability to pay rent, also influence eviction rates and recovery outcomes (24), further threatening both sustainability and program equitability.

Compounding these financial challenges is the uncoordinated and heterogeneous nature of recovery home services as well as a lack of comprehensive national data on them. The overall recovery residence landscape remains poorly defined due to insufficient research (22), likely making it difficult to establish funding priorities and integrate substance use treatment with recovery support services effectively. Additionally, no universal or widely applied measures exist to assess the equitability of recovery home services (25). This is particularly concerning given evidence suggesting that economically vulnerable residents are less likely to afford services (23), potentially exacerbating disparities in access and outcomes.

Related to the uncoordinated national landscape of recovery housing, funding for recovery homes is often temporary, context-specific, and reliant on limited scope grants or loans. For example, SAMHSA’s 2024 Building Communities of Recovery grant provides funding for recovery housing (26), but much of the available support is tied to temporary initiatives such as COVID-19 relief efforts. Another major source of funding provided by SAMHSA is temporary and limited in scope as it allocates block grant funding for start-up loans for recovery residence operators (22). Other initiatives are earmarked for priority populations. For example, in 2023, the Texas Health and Human Services awarded over $30,000,000 in COVID Supplemental Awards to support substance use treatment and recovery services, for emerging adults and pregnant and parenting women (Texas HHSC, 2024). While these initiatives offer temporary relief and much needed support, they do not provide long-term financial security for recovery residences. Given the heterogeneous nature of recovery home services, the lack of comprehensive data assessing their national state, and the temporary, context-specific nature of funding, the sustainability of these essential programs remains under threat. Without standardized regulation, consistent financial mechanisms, and robust research, recovery homes will continue to struggle to provide stable and equitable support to those in need.

While research on recovery home sustainability is needed to address the OUD epidemic, little has been published on the topic. Although some work has examined innovative funding models for Level I homes and differences in social climates between the various recovery home levels (27, 28), few studies have focused directly on sustainability, particularly for Level II and III homes. It is critical to study the sustainability context of Level II and Level III homes specifically because these homes provide services beyond the peer-led Level I homes and therefore need additional funding support. Level II and Level III homes may also be eligible for different sources of funding, such as government-funded grants and healthcare reimbursement. This gap in research is critical considering Dr. Nora Volkow’s 2022 call for expanding MOUD access and retaining individuals in care, which will likely increase demand for recovery homes serving people taking MOUD (29).

Given the ongoing OUD crisis and the promise recovery homes hold, conceptualizing recovery home sustainability is imperative. This study aims to describe factors influencing the sustainability of NARR-affiliate certified, Level II, and Level III recovery residences for people taking MOUD as part of their recovery, such as strategic partnerships and current and future funding. This analysis contributes to the growing body of research on recovery homes highlighting the narratives of those who operate and manage these homes. The study also adds to the broader research on sustaining evidence-based interventions to address substance use issues (30–32).

## 2 Materials and methods

### 2.1 Parent study

This analysis is part of a larger study called *Housing for Opioid MAR Expanded Services (Project HOMES)* funded to expand the capacity and evaluate the effectiveness of NARR-affiliate certified Level II and Level III recovery homes that serve people taking MOUD. There are currently 15 recovery homes (8 homes for women and 7 homes for men) in the Project HOMES network located in El Paso, Midland, San Angelo, Austin, and Houston Texas. Six homes are NARR Level II and nine are NARR Level III. All recovery homes within the network accept residents taking any of the FDA-approved MOUD, including buprenorphine, methadone, and both oral and injectable naltrexone. All homes have developed MOUD policies and procedures to facilitate on-site medication storage and access. Eight recovery residences exclusively serve people taking MOUD while the remaining homes in the network are mixed homes. As of the publication of this paper, Project HOMES has served around 600 individuals in total.

At the start of the project, the majority of homes were newly established homes. These homes did not charge residents rent when they started accepting residents. A few homes were already established homes that transitioned to accepting people taking MOUD. Prior to this transition, there were no residents taking MOUD living in these homes. After the transition, incoming residents taking MOUD were not charged rent. Other residents were required to pay rent unless they received other types of financial assistance offered by the homes. Most homes were independent residential properties housing less than 15 people. All homes were located in communities where with MOUD providers, recovery support service providers, and mutual aid groups meetings were present, however, the availability and capacity of these recovery supports varied by site. More information on the parent study methodology can be found in Wilkerson et al., 2024 (33). This study was approved by UTHealth Houston institutional review board.

### 2.2 Data collection

We collected data in two phases. First, we interviewed 29 house managers, program directors, and operators from 14 separate recovery homes who had enrolled in Project HOMES (the 15th home was not included because it was added to the study after this data collection was completed). This data collection phase lasted between October 2021 and May 2022. Most participants were interviewed on-site during the visits; a few were interviewed virtually at other times using teleconferencing software. Interviews lasted between 60 and 90 min. In-person interviews were recorded using audio recorders, while virtual interviews were recorded with both audio and video. In four cases, we conducted follow-up interviews with participants because these participants had more to share on important topics than the time allotted for. In total, we conducted a total of 33 interviews. These interviews focused on general questions about working in recovery homes for people taking MOUD. Interview guide question domains can be found in the associated study protocol paper (33). Between June and July 2023, we completed 16 additional follow-up interviews among the same study participants to capture additional data about sustainability practices. All of these follow-up interviews were conducted virtually and were recorded using both audio and video. While we crafted the interview questions to capture concepts salient to sustainability, we did not use the term “sustainability” in the interview guide out of concern that participants might be unfamiliar with the concept. Instead, participants were asked about their current experiences managing or operating a recovery home, as well as factors affecting program sustainability, such as partnerships, policies, funding sources, major expenses, relevant sources of information on operational practices, and available resources. Example questions included “What are the major costs of running a recovery residence for people taking MOUD?,” “What do you look for in a potential organization or partner for collaboration?,” and “How do you expect to get the resources you need to keep running your residence?” Recordings were transcribed using a secure transcription service. No incentives were offered to the participants.

### 2.3 Participants

House managers ran the homes on a day-to-day basis and provided peer support to residents. Program directors planned operations and supervised programming. Operators were organization heads and oversaw financing, certification, partnerships, and the business side of operations. Participants for the first phase of data collection were recruited using convenience sampling during site visits conducted by investigators twice a year. For the second phase of interviews, we applied purposive sampling to get an even mix of participants based on gender and recovery home city location. We also ensured we interviewed at least one house manager and one operator from each recovery home that we selected for participation.

### 2.4 Data analysis

Two members of the research team (INZ and DG) coded and analyzed the data. They began this process by coding three sample transcripts from the second phase interviews independently. The coders then met to discuss their independent coding, resolve differences in coding approach, and to develop a provisional codebook. During this process, the coders deliberated and debated coding approaches until coding strategy and application was uniform. The provisional codebook was iteratively revised as it was applied to the data. The researchers then coded all second phase transcripts independently, with each coding about half of the transcripts, with regular check-ins to rectify discrepancies in coding approach through group deliberation, and to refine the codebook as needed. For example, some code titles were changed to better represent concepts over time, and some code definitions changed to more comprehensively and accurately represent coded content. As the senior analyst, INZ reviewed all second-phase interview transcript coding for accuracy and faithfulness to the established coding approach, making additions and corrections where necessary. Following this, INZ coded all 33 transcripts independently from the first phase interviews using the finalized codebook. Then INZ and DG thematically analyzed the data, writing analytical statements for major concepts and consolidating them into preliminary themes. The thematic analysis involved interrogating the data for commonalities, divergences, special cases, and outliers, to generate preliminary themes. Then we showcased the preliminary themes to the larger qualitative analysis team for review and adjusted them as necessary to better reflect the data. When writing the results, we balanced perspectives of the participants and highlighted quotations representing a mix of perspectives covering the range of participant experiences.

## 3 Results

### 3.1 Sample characteristics

Participants worked in an even mix of cities and were evenly split male and female. Most participants in the first phase of data collection were house managers. Professions were evenly mixed for the second phase of interviews. Sample characteristics are displayed in Table 1.

**Table 1:** Participant characteristics.

First phase interview participants		Second phase interview participants
Total	*N* = 29 *N* (%)*	(*N* = 16) *N* (%)*
City		
Austin	8 (27.6%)	4 (25.5%)
Houston	7 (24.1%)	3 (18.8%)
Midland	5 (17.2%)	3 (18.8%)
El Paso	4 (13.8%)	3 (18.8%)
San Angelo	5 (17.3%)	3 (18.8%)
Profession**		
Owners/operators	4 (13.8%)	7 (37.3%)
House managers	18 (62.0%)	6 (31.3%)
Program director	8 (27.6%)	6 (31.3%)
Gender		
Male	14 (55.7%)	9 (56.3%)
Female	15 (48.3%)	7 (43.8%)

*Values may not add up to 100% due to rounding error. **Values do not add up to 100% because some participants served in multiple roles.

### 3.2 Thematic analysis

In this section, we describe three themes that characterize recovery home managers, program directors, and operators’ experiences on topics relevant to the sustainability of recovery homes that house people taking MOUD. Each quotation represents a unique participant, meaning no participant was quoted more than once in the results. The first theme details how recovery home operators rely on grant funding and private contributions to sustain operations. The second theme reveals that participants strategically partnered with external organizations and leveraged professional certifications to maintain operations and help residents succeed. The third theme illustrates that after grant funding ends, many recovery homes will start charging rent which will cause operators to balance costs with service value.

#### 3.2.1 Relying on a mix of private donations and grants

As they reflected on funding availability for the homes, recovery home operators described a complex and demanding funding landscape. Operators and program directors agreed that no single source of funding covered all operational expenses. Operators and program directors often depended on a combination of private donations and grant funding to meet their financial needs. Operators and program directors highlighted that each funding source came with considerations. Private donations gave operators more financial flexibility, but it was risky to rely on charitable donations as those donations were not dependable. Given the mix of grant funding and private donations, it was a challenge to make sure the recovery home was equipped to meet residents’ needs – especially when those needs were as multifaceted as those of people who take MOUD. One program director described:

We kind of just came up with ideas of how to keep us afloat without depending so much on funding. ‘Cause that’s our problem. I don’t know if that’s the house’s problems, but for us it is. Like I said, it’s because of our community. Not everybody has the means… some of them are sick, and some of them have dual diagnosis. They have mental [health] issues that have not been addressed in years. They have abscesses. They have infections. They haven’t been tested for HIV… So, it’s not just the MAT [MOUD] medication program that they’re needing from us. They need everything as a whole. (Program director).

Several operators recalled prior experiences being awarded community or government grant funding, but they characterized this process as uncertain, stressful, and time-consuming. Even when operators were willing and capable of applying for grant funding, they faced challenges to doing so. Drafting a standout application required substantial investment from staff members who have both the time, which is in short supply, and specialized skills, that staff often lack. The application process could be confusing and highly competitive. One operator described approaching a funder:

[Funders would say]…Write up the plan. We'll put it on the list and this sounds like a good [idea] maybe in eight years from now maybe we can get started," you know, all this kinda stuff is what I was hearing. And, you know, says, "Oh, well, this sounds like a great idea but, you know, there's like 88 others that are already on the list wanting to be fund…get funding and so you'll be number 89 and, oh, aren't you cute? (Operator).

All recovery homes featured in this study received funding from Project HOMES through a grant from the Texas Health and Human Services Texas Targeted Opioid Response (TTOR). A major goal of Project HOMES was to increase the capacity of recovery homes in Texas to serve this particularly stigmatized group of people. Through Project HOMES, in some cases, operators were able to house clients who they had not been able to serve before. Participants were clear that Project HOMES funding was essential for establishing recovery homes capable of supporting MOUD recipients. In one case, an operator attributed being able to serve people taking MOUD to the Project HOMES grant because previously MOUD use was not universally welcome or used publicly in recovery homes. This operator said:

I'm really grateful that there is a grant [Project HOMES’s TTOR funding]. You guys are helping these individuals to really recover, because I know prior to this it was kind of, you know, don't ask, don't tell… with the MAT [Medication assisted treatment, commonly known as MOUD] program. And I think there's been some stigma that, you know, people thought, well, you're cheating or you're really not doing recovery. (Operator).

TTOR, as the funder of the Project HOMES grant, required that all homes involved in this study be certified by the local NARR affiliate. NARR certification ensures recovery residences meet established standards for safety, recovery support, and ethical operations. To maintain certification and provide the necessary amenities and services for individuals on MOUD, significant expenses were incurred by the recovery homes, which were covered by the Project HOMES grant. For example, recovery home staff needed to implement secure MOUD storage plans and provide education to prevent medication misuse. Additional costs included staff labor for attending ongoing learning community meetings and engaging with the broader recovery community, crucial for staying updated on MOUD and recovery.

While NARR certification itself had a small cost, the Project HOMES grant covered it and also covered major up front expenses, such as creating communal living spaces and maintaining safety standards like fire extinguishers, smoke alarms, first aid kits, and overdose reversal medication. Fortunately, the Project HOMES grant also covered the start-up costs for managing MOUD in the homes, including staff training and securing MOUD with lock boxes. Because staff were paid through Project HOMES, their ongoing education was also covered. Operators and program directors emphasized that this funding was essential for sustaining homes that supported MOUD, as without it, making the necessary operational changes would have been very difficult.

Having access to TTOR funding expressly to house people taking MOUD appeared to afford the participating recovery homes the capability and clout to take MOUD use out of the shadows. Operator, program director, and house manager narratives across all five cities noted that a one-size-fits-all funding approach did not currently exist, and critical state funding like that from TTOR was needed for long-term sustainability. For each recovery home system, operators harnessed money from several sources to ensure both the organization and residents could survive.

#### 3.2.2 Leveraging strategic partnerships and professional certifications

Several operators and program directors, describing experiences operating without grant-funding, had worked out ways to overcome prohibitive rent costs which would otherwise deny access to people who could not afford it. In some cases, operators had to leverage strategic partnerships to help cover some of the residents’ costs and to extend services. For example, some operators partnered with shelters for the unhoused or services for people with mental health concerns. Those organizations covered a portion of the rent for residents who lacked the ability to pay rent, providing recovery home operators with some dependable income. In one case, an operator reported that the Texas Rent Relief Program helped a recovery home persevere through the COVID-19 pandemic by providing over $20,000 in rent for their residents, which was a critical relationship for sustaining operations through a time when residents could not work or pay rent.

In the context of Project HOMES, participants talked about how partners extended services or improved resident outcomes. For example, a recovery home program director described partnering with a local MOUD clinic to provide hepatitis C and HIV education and testing in-house. Similar partnerships helped provide COVID vaccines to residents during the pandemic. Local mental health services, detox clinics, and MOUD provider partners also referred their housing-insecure clients to this recovery home for residence. Some of these partners paid for residents’ mental health medications. Using memoranda of understanding agreements, other partners provided a share of rental money for their clients who were not part of Project HOMES. House managers were always looking for resources that could help them fill budget shortfalls and had to be resourceful. House managers turned to food pantries and other recover support service organizations for donations so that residents could eat for free. One house manager remarked:

“There’s plenty of food because of people that donate and stuff. Like right now, I don’t think nobody is gonna go hungry in this house. The other day, [another recovery residence] cleaned out their pantry, and they gave us a lot of canned goods, a lot… And [a local recovery support service organization] sometimes will bring stuff for the community. (House manager).

Frequently, recovery house managers took clothing donations, as stocking a wardrobe was typically not within the budget. Some participants said residents experiencing housing insecurity, who were recently incarcerated, or who just left a detox center, needed these local resources the most. Those residents often moved in without funds for clothing, toiletries, or food. House managers and program directors connected residents to these resources, ensuring resident needs were met without costing the recovery home itself. Although these contributions were critical for maintaining quality services to a diverse resident population, they did not directly pay for daily operating costs and were not easily turned into cash.

Partnering with a local NARR affiliate was another critical collaboration for recovery home staff. NARR certification improved long-term sustainability because certification increased participant confidence in service quality and residence safety. Residence safety helped residents feel their living conditions were adequate which improved recruitment and retention, and ultimately program sustainability. Describing how NARR accreditation improved safety, a house manager remarked:

We're very different from other sober livings. We have safety measures. There's checks and balances around the house. It [accreditation] ensures that I’m prepared as a manager because you get asked questions by the accreditor. I feel like we just come with this extra level of professionalism and checks and balances. (House manager).

One operator likened certification to college accreditation, saying that funders liked seeing the stamp of legitimacy before awarding residence with a grant. “It’s sort of like going to college. You want an accredited university before you dump a lot of money into it.” While accreditation was oftentimes time-intensive and sometimes costly, it was worth it to operators for the enhanced legitimacy in an industry where service quality varies tremendously.

Partnering with the local NARR affiliate also helped participants do their jobs more efficiently and effectively. For example, the local NARR affiliate facilitated monthly calls for recovery home staff to learn from each other and the statewide network of recovery homes. This mechanism provided a private and appealing opportunity for recovery home staff to learn from one another how to serve people taking MOUD. Some manager participants mentioned looking forward to the calls and enjoying preparing things to teach their colleagues because the learning opportunities ensured smooth operations and ultimately helped residents.

#### 3.2.3 Post-funding sustainability: charging rent

Project HOMES was funded to expand to the availability of NARR-certified recovery homes in Texas that serve people taking MOUD. Project HOMES also evaluates the effectiveness of these homes. Project HOMES is in its 5^th^ funding year of operations and hopes to renew the grant with TTOR for another funding cycle. When asked how operations would continue after Project HOMES ends, recovery home operators reported that charging rent would be essential to keep doors open. In addition, relying on resident rent money to continue operations would increase pressure to fill beds and make high resident turnover a threat to sustainability. To attract and keep tenants, operators, program directors, and house managers demonstrated their value to the community and those living in their residences. This involved educating community members, stakeholders, and potential clients about how their recovery homes helped residents stabilize their lives, find good jobs, contribute to society, and ultimately stay in recovery. While charging rent may become inevitable when funding through Project HOMES ends, operators and program directors were clear that equity and access to services would be sacrificed. Many people facing high barriers to accessing recovery home services would be left out due to a future rent requirement. Participants strongly believed that their homes should welcome anyone committed to recovery. Yet, they acknowledged that requiring residents to pay rent would disproportionately exclude marginalized groups such as people with low incomes and people of color. This condition represented a dilemma for many operators and program directors. One operator’s views were shaped by his own experience in recovery, during which he was surrounded by a diverse group of other people in early recovery. This operator observed that diversity enhances the recovery process:

When you eliminate those financial hurdles…I think it’s almost an ideal model for people to cohabitate and learn from one another, and for them to really sort of rub elbows. You know, the Buddhists have a saying, the rocks polish each other by their interactions. And I think we have a pretty wide mix of people that are polishing each other that are creating friendships and recovery bonds and that are pretty amazing to see. (Operator).

Recalling experiences running recovery homes before receiving TTOR funding, two operators referred to the “Goldilocks dollar amount,” a price point where operational costs were met, but at the same time, where most people seeking recovery support could afford it. In some cases, before receiving TTOR funding, operators offered sliding-scale rent frameworks to ensure the maximum number of people could afford their services while remaining financially solvent. In general, participants viewed that charging people in recovery rent, especially those with complex needs like people taking MOUD, was inequitable. Participants struggled to articulate a great system for keeping costs to residents low while maintaining high-quality services in lieu of major grant funding or private donations. With such limited budgets, operators were sometimes uncertain about how they would sustain operations for residents taking MOUD after Project HOMES ends. One operator commented:

I don’t know that I would be able to sustain a MAT [MOUD]-only home. With any type of quality, the way that we do it. I mean, any Joe Schmo can go rent a house, put some furniture in it, put some utilities on it, and go out there to the community and talk about they got a sober living house, and they’ll provide little or no oversight to it. Or if they do provide good oversight to it, well, they’re needing to get paid for that. (Operator).

Participants talked about ways they planned ahead and creatively saved costs to ensure high value at sustainable operational costs. Managers and program directors created weekly or monthly house budgets and sometimes made tough decisions about what to spend on to ensure smooth operations and comfortable environments for residents, for example, whether and when to replace worn furniture or what supplies should be restocked. On budget planning, one house manager remarked:

[We talk about] if we need to change things about the grocery shopping… We talk about supplies and how we’re doing on those. We talk about if there’s any, [issues] like… We think my refrigerator is going out, are we gonna need to replace it, or can it be repaired? I mean there’s just so many different things [to budget for] in the home. (House manager).

While carrying out operations after Project HOMES concludes will be difficult, most participants expressed a deep commitment to continuing operations and serving residents who take MOUD.

## 4 Discussion

### 4.1 Discussion of findings

In this analysis, we identified themes related to recovery home funding sources and organizational relationships that improve the sustainability of recovery homes. While other published research has discussed the sustainability concerns of recovery homes with different funding structures and services provided (6), our research is the first to analyze the sustainability of Level II and Level III recovery homes for individuals taking MOUD as part of their recovery journey. Given that recovery homes show promise in addressing the mounting opioid crisis (6), ensuring their sustainability is crucial. This study described how recovery home stakeholders sustained operations highlighting their own narratives on the topic.

Our findings reveal that NARR-affiliated, grant-funded, Level II and Level III recovery homes operators often relied on a patchwork of funding opportunities, each with conditions and considerations, to stay in business. Notably, we found that once TTOR funding concludes, most participating operators plan on making up budget shortfalls by charging residents rent. Operators were concerned with how the tension between keeping costs low and maintaining adequate services may affect service equity. With increased rent, program equitability and therefore resident outcomes may suffer. Underscoring these concerns, recent recovery support service stakeholders have drawn attention to systemic racism as a paramount concern in effectively fighting the OUD epidemic, highlighting the need for research on racial equity in this context (34). Our findings suggest that rent-funded recovery home models may ultimately deny services to some residents from marginalized groups. This is an important finding considering many recovery residences struggle with financial stability (6) and are commonly dependent on resident rent rather than major federal funding or healthcare reimbursement to sustain services (21). To ensure service equity, we recommend long-term, increased government funding, large-scale public housing initiatives, and more research on building the capacity and equity of recovery homes. We recommend increasing state and local funding for recovery home financing and sustainability factors, including improved recovery support service infrastructure and more opportunities for stakeholder networking and collaboration. Recovery home operators should enhance their internal capacity to secure such funding through training and partnerships with academic organizations.

Recovery home sustainability is especially under threat in shifting political or economic environments. For example, a study examining the conditions of 1,342 recovery homes during the early days of COVID-19 found that 8% of the residences were at risk for closing (23). Recovery homes serving more economically vulnerable residents were more likely to close, probably because these residents were more likely to lose income during the pandemic (23). Recovery homes relying on local contracts for funding were also more likely to close during the pandemic (23). Recovery homes relying more on private donations were slightly less likely to close, suggesting that diversified funding may increase sustainability (23). These findings suggest that reliance on resident rent reduces program sustainability and that more federal funding may improve it. The potential inequities associated with self-pay recovery support services are particularly concerning, given the current national political landscape where the role, size, and budget of the federal government is under severe scrutiny. Cornerstone safety net services like The Affordable Care Act and Medicaid, evidence-based public health policies recently bolstered by the American Rescue Plan Act and the Families First Coronavirus Response Act, are now under serious threat of being cut (35).

State and federal agencies should consider ways to strengthen support and funding, especially during vulnerable periods like national disasters such as COVID-19 or political environments where the value of evidence-based social services is questioned. The Medicaid and CHIP Payment and Access Commission found that while Medicaid finances a range of recovery support services, including peer support, skills training, and supported employment, recovery supportive housing remains one of the least commonly covered services (36). Recovery supportive housing is similar to the recovery homes discussed in this analysis insofar as it houses people with an SUD, but the service is specifically aimed at providing housing and wraparound social services to chronically unhoused and/or disabled people who also have an SUD (36). Only four states have leveraged innovative Medicaid reimbursement approaches for recovery-supportive housing (36). Expanding Medicaid’s role in covering recovery-supportive housing could substantially reduce barriers for chronically unhoused and/or disabled people with an SUD seeking stable, supportive environments for recovery. Recovery advocacy organizations can play a critical role in advancing policies that provide Medicaid reimbursement and insurance coverage for living in recovery-supportive housing homes, ensuring that these essential services remain available to those who need them most. Advocates can build on this model to advance policies that might cover recovery homes like the ones featured in this analysis with Medicaid reimbursement, too, dramatically expanding housing opportunities for people who are working on recovery.

Leveraging partnerships with other recovery and social services emerged as an important strategy for maintaining operations and extending services. Participants valued calls with other Project HOMES-affiliated recovery homes, which offered frequent opportunities to share critical and timely skills and information. Participants also appreciated partnering with a broad network of academic partners, state agencies, and other recovery home stakeholders or first learning how to serve residents that take MOUD. Recovery home stakeholders found a broader network of affiliates and partners beneficial for the sustainment of services. These findings emphasize the unique challenges faced by Level II and Level III homes as they provide more services and have higher operational costs lower-level homes or homes that did not follow NARR-affiliate standards. Other researchers have highlighted communicating with other recovery home partners, local leaders, and businesses and as key strategies to garner support (6). Recovery home stakeholders should consider ways to strengthen partnership communication and co-education to rapidly share information and provide hands-on, peer-led training to professionals in the field. Recovery home stakeholders can help economically vulnerable residents and sustain recovery homes by strengthening partnerships sharing information on efficient operations and funding strategies with partners.

Continued and expanded partnerships with academic institutions can also help recovery homes serving people taking MOUD to sustain government-sponsored research funding. These partnerships can also sustain operations by providing cutting-edge technical assistance. For example, the Missouri Department of Mental Health administered similar grants funded by the State Targeted Response (37). This grant required recovery home recipients to be deemed “friendly” to people taking MOUD by the Department of Mental Health. Other states can consider allocating funds to sustain recovery homes and improve access for people taking MOUD. A recent survey of recovery home stakeholders demonstrated that the number one technical assistance and training topic of interest was sustainability planning (38). Academic institutions can help recovery homes get grant funding, train recovery home staff in grant writing, and assist them with program planning and evaluation.

Each participant we interviewed recognized the benefit and importance of partnering with the local NARR affiliate and obtaining certification for resident safety, technical assistance, and appealing to funders. Operators also relied on the support and legitimacy associated with NARR-affiliate certification build trust with residents and help raise money. Furthermore, partner calls with other NARR-affiliate certified recovery home staff helped organizations share information and learn from each other. An important political condition to consider in context with our findings is the passage of Texas House Bill 299 in the 88th Texas Legislature in 2023. This law will require that all recovery homes be certified by the Texas NARR affiliate beginning in 2025 to receive state funding (39). While each participating organization in this analysis was already required to be certified, this law, along with similar legislation in other states, will require other recovery home agencies to get certified if they want to receive future state funding. This new policy could increase service homogeneity, consistency, and external political and funding support. To facilitate recovery home certification, government agencies should consider financially assisting recovery home operators who cannot pay certification costs.

### 4.2 Limitations

While this analysis is among the first to investigate the sustainability of NARR-affiliated Level II and Level III recovery homes for people taking MOUD (i.e., recovery homes with a designated house manager and additional recovery support services designed for people taking MOUD), it has limitations. First, we did not randomly select participants. However, to account for nonrandom sampling, we interviewed at least one manager and one operator from every recovery home involved in the larger study to diversify the contributing voices and capture broad perspectives. Another limitation is that the interview data may be subject to social desirability bias given that interviews were conducted by the Project HOMES team. Our project provides a significant amount of funding to the recovery homes studied. As a result, participants may have answered questions in ways they thought we would like to hear, instead of responding objectively. Similarly, social desirability bias may have affected participant responses surrounding the topic of NARR-affiliate certification, as recovery homes in the study are required to be certified and subsequently adhere to the NARR-affiliate standards. To account for this potential bias, we assured participants their identifiable information was protected, and that open and honest responses were desired. Additionally, we did not formally and quantitatively capture demographic information from participants such as number of years of work experience, or whether the participant had personal experience with an OUD or taking MOUD. Future studies may collect this information to add more robust detail to the qualitative data. Lastly, readers should apply our qualitative research findings to understand other contexts carefully, taking into consideration the timing, setting, and conditions the cases these findings are compared to.

## 5 Conclusion

Our study leveraged the voices of recovery residence stakeholders to identify important factors for sustaining NARR-affiliate certified Level II and Level III recovery homes serving people taking MOUD as part of their recovery. Recovery home sustainability depended on securing diverse funding sources, but each funding source came with unique considerations and constraints. Recovery home stakeholders leveraged their partnerships and certifications ensure smooth operations and secure funding. Charging resident rent will be a primary source of funding once state funding concludes, but relying on resident rent may come with a cost to service equity, as economically disadvantaged residents may have difficulty keeping up with rent payments, especially when times are tough. Unfortunately, it appears that service equity and sustainability are in tension when operations rely on resident rent, as residents capable of paying rent are likely to start off with more privilege. Balancing service equity and sustainability will be a crucial concern for recovery home stakeholders as they navigate sustaining operations after grant funding concludes.

To increase recovery home sustainability, stakeholders can strengthen professional networks and partnerships and leverage their partners’ experiences with fundraising and lowering operational costs. Policymakers can support recovery homes by establishing long-term funding mechanisms and reducing financial barriers to professional certification, ultimately, improving service quality and access. Researchers can contribute to recovery home sustainability by lending their skills in strategic planning, program evaluation, and grant writing to recovery home operators to assist with funding and sustaining services. Future researchers should also quantitatively assess a comprehensive set of recovery homes across the country for sustainability to understand more concretely the breadth and depth of industry stability. Such efforts should be guided by contemporary sustainability models to ensure relevant indicators are queried. The results of such research can shape how researchers and practitioners conceptualize sustainability for this unique recovery support service and therefore guide where future resources should be directed. By increasing recovery home sustainability, stakeholders, researchers, and policymakers can support more people to recover from OUD and, ultimately, save lives. Sustaining recovery homes is critical to fighting the unfolding OUD epidemic, and the strategies and considerations outlined in this research can help ensure recovery homes remain viable and effective recovery support services for people in need.

## Data Availability

The data presented in this article is not available to the public because data may contain personally identifiable information even if names and places are redacted. Participants were assured we would not share outside the study team. Requests to access the datasets should be directed to sheryl.a.mccurdy@uth.tmc.edu.
